# Ocular surface oxidative stress, MMP-9, and corneal neuroimmune alterations in allergic conjunctivitis with dry eye: a cross-sectional study

**DOI:** 10.3389/fmed.2026.1942693

**Published:** 2026-09-08

**Authors:** Kang Yu, Jingjing Xu, Yanqing Li, Jiale Li, Di Wu, Haole Zhu, Yifeng Yu

**Affiliations:** Department of Ophthalmic Center, The Second Affiliated Hospital, Jiangxi Medical College, Nanchang University, Nanchang, Jiangxi, China

**Keywords:** allergic conjunctivitis, corneal nerves, dry eye, ocular surface barrier, oxidative stress, tear biomarkers

## Abstract

**Purpose:**

To investigate the associations among ocular surface oxidative stress, MMP-9–associated barrier dysfunction, and corneal nerve alterations in patients with allergic conjunctivitis-associated dry eye.

**Methods:**

In this cross-sectional study, participants were classified into healthy controls (HC), patients with allergic conjunctivitis (AC), and patients with AC-associated dry eye (AC + DE). Ocular surface symptoms and signs, tear film function, corneal fluorescein staining, and *in vivo* confocal microscopy (IVCM) parameters were evaluated. Tear malondialdehyde (MDA), superoxide dismutase (SOD) activity, and matrix metalloproteinase-9 (MMP-9) concentrations were measured. Spearman correlation analyses were performed in patients with AC and AC + DE, and receiver operating characteristic (ROC) curve analyses were used to evaluate the ability of tear biomarkers to distinguish AC + DE from AC alone.

**Results:**

Compared with patients with AC alone, those with AC + DE exhibited higher Ocular Surface Disease Index scores, shorter tear film break-up time, lower Schirmer I test values, and more severe corneal fluorescein staining (all *P* < 0.001). IVCM further demonstrated reduced corneal nerve parameters and increased dendritic cell density in the AC + DE group. Tear MDA and MMP-9 concentrations were significantly higher, whereas SOD activity was lower, in patients with AC + DE. Exploratory correlation analyses identified associations among oxidative stress markers, MMP-9, and selected ocular surface parameters. The areas under the ROC curves for MDA, SOD, and MMP-9 were 0.822, 0.730, and 0.789, respectively.

**Conclusions:**

Patients with AC + DE exhibited greater oxidative stress imbalance, higher MMP-9 levels, impaired tear film function, and more pronounced corneal neuroimmune alterations than patients with AC alone. The observed relationships among oxidative stress markers, MMP-9, and tear film parameters suggest that these abnormalities may be interconnected within the AC + DE phenotype. Tear oxidative stress–related biomarkers may also help distinguish AC + DE from AC alone and warrant further evaluation in longitudinal studies.

## 1 Introduction

Allergic conjunctivitis (AC) is among the most common IgE-mediated inflammatory diseases of the ocular surface. It typically presents with itching, conjunctival hyperemia, tearing, and foreign body sensation and can substantially impair quality of life (1). Clinical studies indicate that approximately 31%–36% of patients with AC also meet objective diagnostic criteria for dry eye, making dry eye a frequent ocular surface comorbidity in this population. These patients may present with tear film instability, reduced tear secretion, corneal epithelial damage, persistent ocular discomfort, and fluctuating visual symptoms (2, 3). Dry eye, however, does not occur in all patients with AC. Even among patients with comparable allergic manifestations, the extent of ocular surface damage and the eventual clinical outcome may differ considerably. This variability suggests that AC-associated dry eye represents a heterogeneous clinical phenotype, the underlying mechanisms of which remain unclear (4).

Most studies of AC have focused on well-established mechanisms such as IgE-mediated allergic responses, mast cell activation, and T helper 2 (Th2)-dominant inflammation (1, 5). Although these processes explain much of the initiation and persistence of allergic inflammation, they do not fully account for differences in ocular surface involvement between patients (6). Oxidative stress has increasingly been recognized as a possible link between chronic ocular surface inflammation and tissue injury. Sustained inflammatory stimulation can promote excessive production of reactive oxygen species (ROS), leading to amplification of inflammatory signaling, lipid peroxidation, weakened antioxidant defenses, and disruption of ocular surface redox homeostasis (7, 8). Matrix metalloproteinase-9 (MMP-9), a biomarker associated with ocular surface barrier dysfunction, can degrade epithelial tight junction proteins and thereby compromise epithelial integrity and tear film stability. Changes in the ocular surface microenvironment may also be accompanied by alterations in the corneal nerve plexus, which have been reported in dry eye and other chronic ocular surface disorders (9–11). Taken together, these observations raise the possibility that oxidative stress, barrier dysfunction, and corneal nerve changes are involved in the development of dry eye in a subset of patients with AC. This possibility, however, has not been systematically examined in a clinical population.

In this cross-sectional observational study, we compared clinical ocular surface findings, corneal nerve microstructure, tear oxidative stress markers, and ocular surface barrier–related biomarkers among healthy controls, patients with AC alone, and patients with AC-associated dry eye (AC + DE). We also examined the relationships among these parameters and assessed whether tear biomarkers could distinguish AC + DE from AC alone.

## 2 Materials and methods

### 2.1 Study design and participants

Participants were consecutively recruited for this cross-sectional observational study at the Department of Ophthalmology, the Second Affiliated Hospital of Nanchang University, between September 2025 and April 2026. The study protocol was approved by the Medical Ethics Committee of the Second Affiliated Hospital of Nanchang University [Approval No. O-Medical Research Ethics (2025) 162] and was conducted in accordance with the Declaration of Helsinki. Written informed consent was obtained from all participants or their legal guardians before enrollment.

A total of 90 participants aged 10–60 years were enrolled and classified into three groups: healthy controls (HC, *n* = 30), patients with allergic conjunctivitis (AC, *n* = 30), and patients with allergic conjunctivitis-associated dry eye (AC + DE, *n* = 30). Healthy controls were recruited from healthy volunteers at the same institution during the study period and had no history or clinical evidence of allergic conjunctivitis, dry eye, or other ocular surface diseases. Skin prick testing and serum allergen-specific IgE testing were not routinely performed in healthy controls. Allergic conjunctivitis was diagnosed according to typical allergic symptoms and signs together with a positive skin prick test or serum allergen-specific IgE test (12). Only patients with seasonal or perennial allergic conjunctivitis were included. Among patients with AC, those with TBUT ≥10 s and a CFS score ≤1 were classified as the AC group, whereas those with TBUT <10 s and/or CFS ≥2 were classified as the AC + DE group. Because ocular symptoms attributable to AC and dry eye overlap substantially, OSDI was not used as the primary criterion for group allocation. Nevertheless, all patients classified as AC + DE had an OSDI score ≥13, indicating the presence of clinically relevant ocular symptoms in addition to objective ocular surface abnormalities. Thus, the AC + DE group fulfilled both symptom-based and objective components consistent with the TFOS DEWS II diagnostic framework (13). Participants were excluded if they had worn contact lenses within the previous 3 months; undergone ocular surgery or experienced ocular trauma within the previous 6 months; had active ocular infection, other ocular surface diseases, autoimmune diseases, or diabetes mellitus; were pregnant or breastfeeding; or had received topical corticosteroids, topical immunosuppressants, topical anti-allergic medications, or systemic antihistamines within the previous month.

All participants underwent demographic data collection, clinical ocular surface examinations, tear sample collection, and *in vivo* confocal microscopy (IVCM). Analyses were performed using data from a single study eye. If both eyes met the eligibility criteria, the eye with the higher ocular itching score was selected. If the ocular itching scores were identical between the two eyes, the right eye was included.

### 2.2 Clinical ocular surface examinations

All participants underwent a comprehensive ocular surface evaluation, including completion of the OSDI questionnaire (14), assessment of ocular symptoms and signs, TBUT, the Schirmer I test, and CFS (15). Ocular itching, conjunctival hyperemia, and papillary reactions were graded using previously described scoring systems. Ocular itching was scored on a 5-point scale (0–4), whereas conjunctival hyperemia and papillary reactions were each graded on a 4-point scale (0–3), with higher scores indicating greater severity (12). TBUT was measured three consecutive times, and the mean value was used for analysis. The Schirmer I test was performed without topical anesthesia for 5 min. Corneal fluorescein staining was assessed according to the Oxford grading scheme (16). All examinations were performed in a standardized sequence by the same experienced ophthalmologist.

### 2.3 Tear sample collection and biomarker measurements

Unstimulated tear samples were collected from the study eye using sterile microcapillary tubes before ocular surface examinations and corneal fluorescein staining. Approximately 10–20 μL of tears was collected from the inferior tear meniscus of each participant. Samples were immediately transferred to sterile microcentrifuge tubes and stored at −80 °C until analysis. Care was taken to avoid contact with the corneal or conjunctival surface during collection.

Tear malondialdehyde (MDA) concentrations and superoxide dismutase (SOD) activity were determined using commercially available thiobarbituric acid reactive substances (TBARS) and water-soluble tetrazolium-1 (WST-1) assay kits (Solarbio, Beijing, China), respectively, according to the manufacturers' instructions. Tear MMP-9 concentrations were quantified using a human sandwich enzyme-linked immunosorbent assay (ELISA) kit (Abcam, Cambridge, UK). All measurements were performed in duplicate, and the mean value was used for statistical analyses.

### 2.4 *In vivo* confocal microscopy

Corneal IVCM examinations were performed using the Heidelberg Retina Tomograph III equipped with the Rostock Cornea Module (HRT III/RCM; Heidelberg Engineering, Heidelberg, Germany) (17). Before image acquisition, topical anesthesia was achieved using 0.4% oxybuprocaine hydrochloride eye drops. Consecutive scans were obtained from the central cornea and the inferior whorl region. According to previously published protocols (17), three representative, well-focused, non-overlapping images without motion artifacts were selected from each region for quantitative analysis. Corneal nerve parameters, including corneal nerve fiber density (CNFD), corneal nerve branch density (CNBD), corneal nerve fiber length (CNFL), corneal total branch density (CTBD), corneal nerve fiber area (CNFA), corneal nerve fiber width (CNFW), and corneal nerve fractal dimension (CFracDim), were quantified automatically using ACCMetrics software (University of Manchester, Manchester, UK) (18). Dendritic cell (DC) density in the central cornea and inferior whorl was quantified manually using ImageJ software (National Institutes of Health, Bethesda, MD, USA) (19). Image analyses were performed independently by two masked observers. Any discrepancies were resolved by joint review until consensus was reached.

### 2.5 Statistical analysis

All statistical analyses were performed using SPSS Statistics version 27.0 (IBM Corp., Armonk, NY, USA), and figures were generated using GraphPad Prism version 10.0 (GraphPad Software, San Diego, CA, USA). Data distribution was assessed using the Shapiro–Wilk test. Normally distributed variables are presented as the mean ± standard deviation (SD) and were compared using one-way analysis of variance (ANOVA) followed by Tukey's *post hoc* test. Variables that were not normally distributed are presented as the median [interquartile range (IQR)] and were analyzed using the Kruskal–Wallis test followed by Dunn's multiple-comparison test. Categorical variables were compared using the *χ*² test or Fisher's exact test, as appropriate. Spearman's rank correlation analysis was used to evaluate associations between variables in the pooled AC and AC + DE cohort. *P* values for the correlation matrix were adjusted for multiple comparisons using the Benjamini–Hochberg false discovery rate (FDR) procedure. As sensitivity analyses, Spearman correlations were repeated separately within the AC and AC + DE groups, and partial Spearman correlations were calculated in the pooled cohort after adjustment for group status. Receiver operating characteristic (ROC) curve analysis was performed to assess the ability of tear biomarkers to distinguish patients with AC + DE from those with AC alone. Optimal cutoff values were determined using the maximum Youden index (sensitivity + specificity−1), with the corresponding sensitivity and specificity reported. All statistical tests were two-sided, and *P* < 0.05 was considered statistically significant; for the correlation matrix, significance was based on FDR-adjusted *P* values.

## 3 Results

### 3.1 Baseline characteristics

A total of 90 participants were included in the study, comprising 30 HC, 30 patients with AC, and 30 patients with AC + DE. Their demographic and baseline clinical characteristics are summarized in Table 1. No significant differences were observed among the three groups in age, sex, body mass index (BMI), smoking status, or daily screen time (all *P* > 0.05). Likewise, the AC and AC + DE groups were comparable with respect to AC subtype, the prevalence of allergic rhinitis, and disease recurrence (all *P* > 0.05), indicating that baseline characteristics were well balanced between groups.

**Table 1:** Demographic and baseline clinical characteristics of the study participants.

Characteristic	HC (*n* = 30)	AC (*n* = 30)	AC + DE (*n* = 30)	F/H/*χ*^2^	*P*-Value
Age, years	27.17 ± 7.18 (16–41)	27.40 ± 7.19 (17–40)	27.10 ± 6.85 (17–41)	0.013^1^	0.987
Participants aged <18 years, *n* (%)	2 (6.7%)	1 (3.3%)	1 (3.3%)		
Sex, *n* (%)				1.448^2^	0.485
Male	14 (46.67)	12 (40.00)	10 (33.33)		
Female	16 (53.33)	18 (60.00)	20 (66.67)		
BMI, kg/m2	22.89 ± 2.20	21.85 ± 2.41	22.22 ± 2.04	1.700^1^	0.189
Smoking status, *n* (%)				1.438^2^	0.487
Yes	7 (23.33)	5 (16.67)	9 (30.00)		
No	23 (76.67)	25 (83.33)	21 (70.00)		
Daily screen time, h/day	4.68 ± 1.42	4.30 ± 1.44	4.90 ± 1.37	1.570^1^	0.214
AC subtype, *n* (%)					
SAC	-	21 (70.0)	16 (53.3)	1.800^2^	0.180
PAC	-	9 (30.0)	14 (46.7)		
Allergic rhinitis, *n* (%)	-	13 (43.3)	11 (36.7)	0.278^2^	0.598
Disease episodes, *n* (%)				0.681^2^	0.409
First episode	-	11 (36.7)	8 (26.7)		
recurrent	-	19 (63.3)	22 (73.3)		

Data are presented as mean ± standard deviation (SD), or as *n* (%), as appropriate. HC, healthy controls; AC, allergic conjunctivitis; AC + DE, allergic conjunctivitis-associated dry eye; BMI, body mass index. Superscript numerals indicate the statistical tests used: ^1^one-way analysis of variance (ANOVA); ^2^chi-square test. A two-sided *P* < 0.05 was considered statistically significant.

### 3.2 Clinical ocular surface findings

Clinical ocular surface findings are summarized in Table 2. Compared with healthy controls, both the AC and AC + DE groups had significantly higher OSDI scores, ocular itching scores, conjunctival hyperemia scores, and papillary scores (all *P* < 0.001). Compared with patients with AC alone, those with AC + DE exhibited higher OSDI scores, more severe conjunctival hyperemia, shorter TBUT, lower Schirmer I test values, and higher CFS scores (all *P* < 0.001). Ocular itching and papillary scores did not differ significantly between the AC and AC + DE groups (both *P* > 0.05).

**Table 2:** Clinical ocular surface parameters among the study groups.

Clinical parameters	HC (*n* = 30)	AC (*n* = 30)	AC + DE (*n* = 30)	*F/H/χ^2^*	*P*-Value
OSDI	6.47 ± 2.71^a^	20.27 ± 6.14^b^	35.97 ± 9.62^c^	152.19^1^	<0.001*
Itching score	0 (0–0)^a^	2 (2–3)^b^	2 (2–3)^b^	61.15^2^	<0.001*
Conjunctival hyperemia	0 (0–0)^a^	2 (1–2)^b^	2 (2–3)^c^	63.52^2^	<0.001*
Papillary score	0 (0–0)^a^	1 (1–2)^b^	2 (1–2)^b^	59.49^2^	<0.001*
TBUT	12.29 ± 1.91^a^	11.50 ± 1.20^a^	5.63 ± 1.39^b^	169.30^1^	<0.001*
Schirmer I test (mm/5 min)	13.60 ± 2.72^a^	13.20 ± 2.44^a^	6.03 ± 2.08^b^	91.69^1^	<0.001*
CFS	0 (0–0)^a^	0 (0–0)^a^	2 (2–2)^b^	70.17^2^	<0.001*

Data are presented as mean ± standard deviation (SD) for normally distributed variables and median [interquartile range (IQR)] for non-normally distributed variables. HC, healthy controls; AC, allergic conjunctivitis; AC + DE, allergic conjunctivitis-associated dry eye; OSDI, Ocular Surface Disease Index; TBUT, tear break-up time; CFS, corneal fluorescein staining. Values sharing the same superscript letter are not significantly different, whereas values with different superscript letters differ significantly (*P* < 0.05). Superscript numerals indicate the statistical tests used: ¹one-way analysis of variance (ANOVA); ²Kruskal–Wallis test. A two-sided *P* < 0.05 was considered statistically significant.

* indicates statistical significance at P < 0.05.

### 3.3 Oxidative stress and ocular surface barrier–related biomarkers

**Figure 1:**
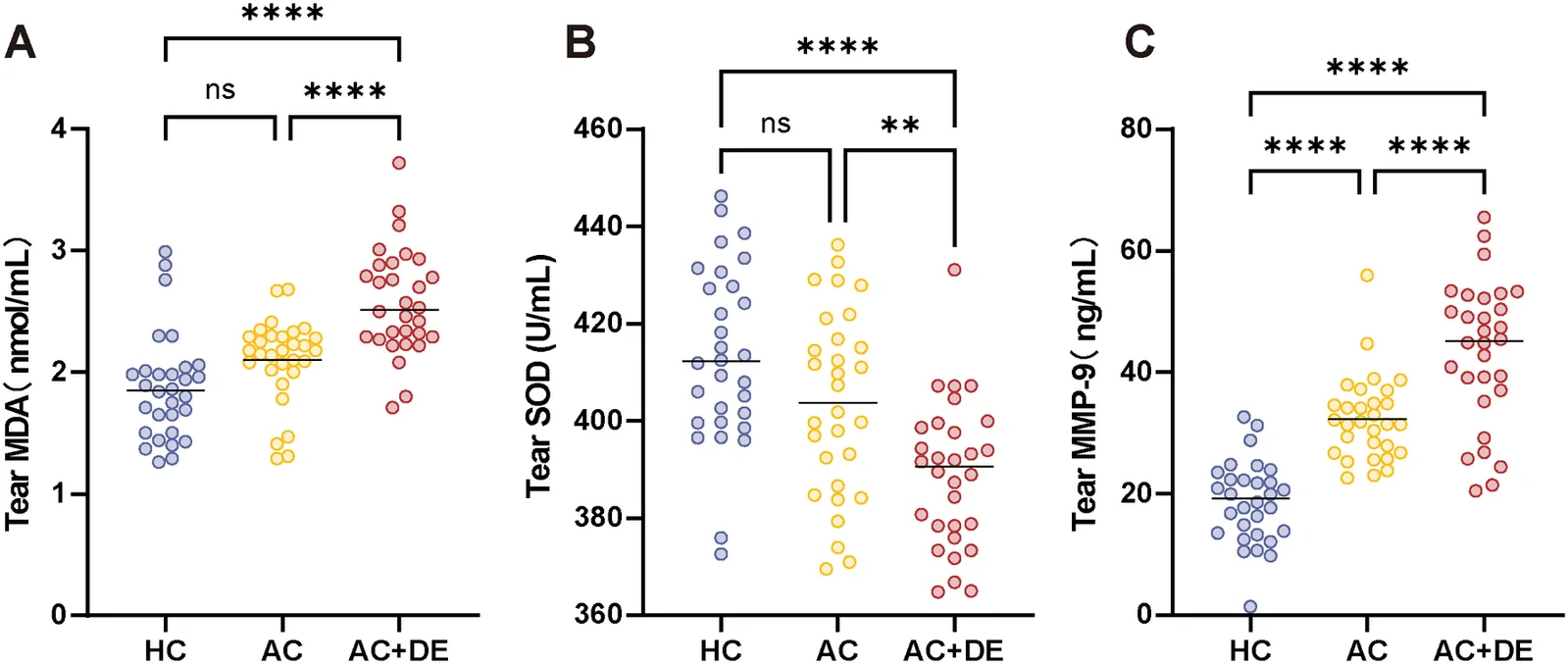
Tear oxidative stress markers and MMP-9 concentrations in the three study groups. **(A)** Tear malondialdehyde (MDA) concentrations, **(B)** tear superoxide dismutase (SOD) activity, and **(C)** tear matrix metalloproteinase-9 (MMP-9) concentrations in healthy controls (HC), patients with allergic conjunctivitis (AC), and patients with allergic conjunctivitis-associated dry eye (AC + DE). Data are presented as mean ± standard deviation (SD). Statistical comparisons were performed using one-way analysis of variance (ANOVA) followed by Tukey's multiple-comparison test. ns, not significant; ns, not significant; *P < 0.05; **P < 0.01; ***P < 0.001; ****P < 0.0001.

Levels of tear oxidative stress markers and ocular surface barrier-related biomarkers are presented in Figure 1. Tear MDA concentrations were significantly higher in the AC + DE group than in both the HC and AC groups, whereas no significant difference was observed between the HC and AC groups. Similarly, tear SOD activity was significantly lower in the AC + DE group than in the other two groups, with no significant difference between the HC and AC groups. Tear MMP-9 concentrations increased progressively from the HC group to the AC group and the AC + DE group, and all pairwise comparisons reached statistical significance.

### 3.4 IVCM assessment of corneal neuroimmune alterations

**Figure 2:**
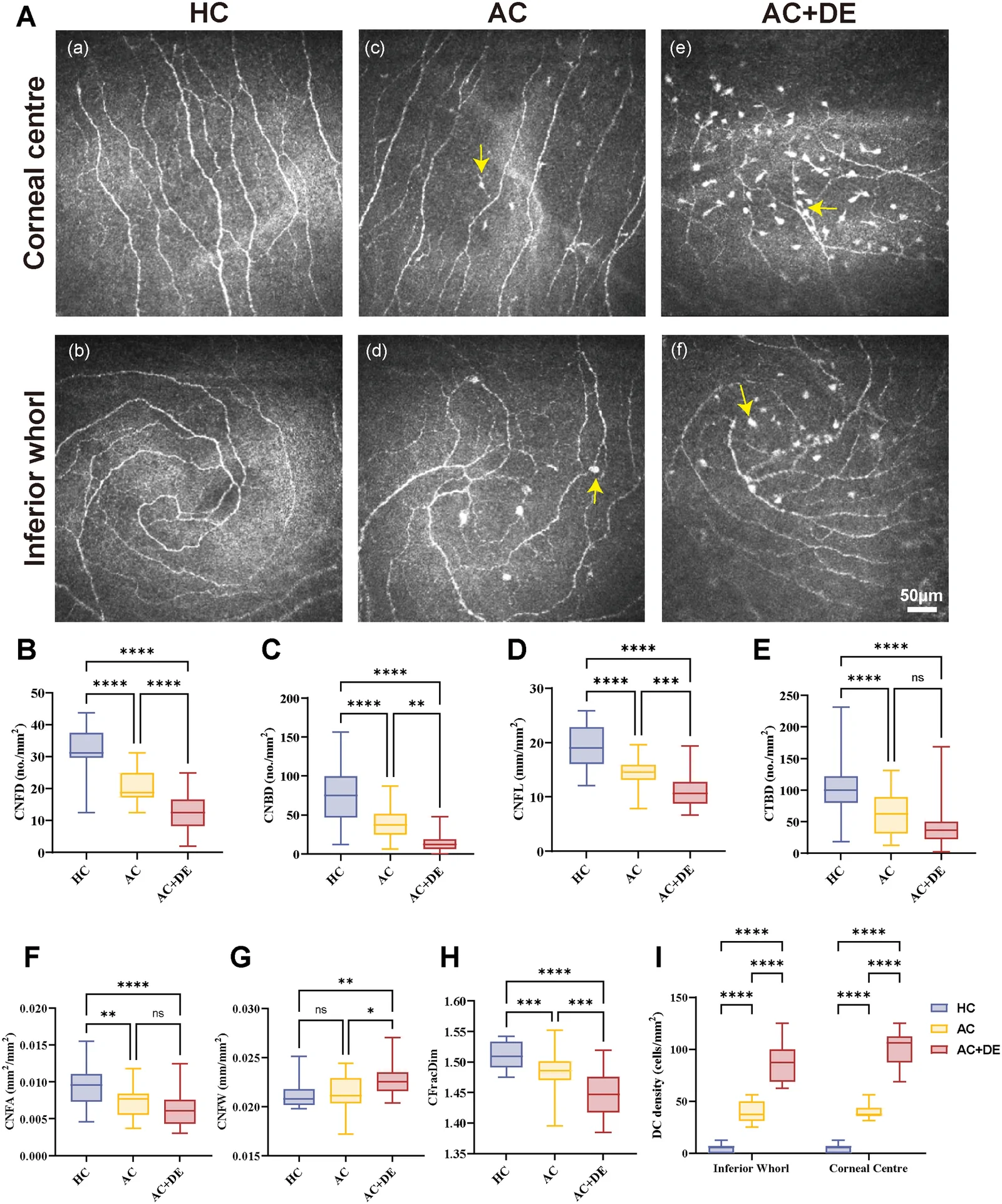
Corneal neuroimmune alterations assessed by *in vivo* confocal microscopy (IVCM). **(A)** Representative IVCM images of the central cornea and inferior whorl from healthy controls (HC), patients with allergic conjunctivitis (AC), and patients with allergic conjunctivitis-associated dry eye (AC + DE). Yellow arrows indicate dendritic cells (DCs). Scale ba*r* = 50 μm. **(B–I)** Quantitative analysis of corneal nerve fibre density (CNFD), corneal nerve branch density (CNBD), corneal nerve fibre length (CNFL), corneal total branch density (CTBD), corneal nerve fibre area (CNFA), corneal nerve fibre width (CNFW), corneal nerve fractal dimension (CFracDim), and dendritic cell (DC) density in the inferior whorl and central cornea. Data are presented as mean ± standard deviation (SD) or median [interquartile range (IQR)], as appropriate. Statistical comparisons were performed using one-way analysis of variance (ANOVA) followed by Tukey's multiple-comparison test or the Kruskal–Wallis test followed by Dunn's multiple-comparison test, as appropriate. ns, not significant; ns, not significant; *P < 0.05; **P < 0.01; ***P < 0.001; ****P < 0.0001.

Representative IVCM images of the central cornea and inferior whorl are shown in Figure 2A. Compared with healthy controls, patients with AC exhibited reduced corneal nerve fibers and increased dendritic cell (DC) density. These changes were more pronounced in patients with AC + DE. Quantitative analyses are presented in Figures 2B–I. Both the AC and AC + DE groups showed significantly lower corneal nerve fiber density (CNFD), corneal nerve branch density (CNBD), corneal nerve fiber length (CNFL), corneal total branch density (CTBD), corneal nerve fiber area (CNFA), and corneal nerve fractal dimension (CFracDim) than the HC group (all *P* < 0.05). Several corneal nerve parameters were further reduced in the AC + DE group compared with the AC group. Corneal nerve fiber width (CNFW) was significantly lower in the AC + DE group than in both the HC and AC groups (both *P* < 0.05). In addition, dendritic cell density in both the central cornea and inferior whorl increased progressively from the HC group to the AC group and the AC + DE group, with significant differences observed for all pairwise comparisons.

### 3.5 Correlation analyses

**Figure 3:**
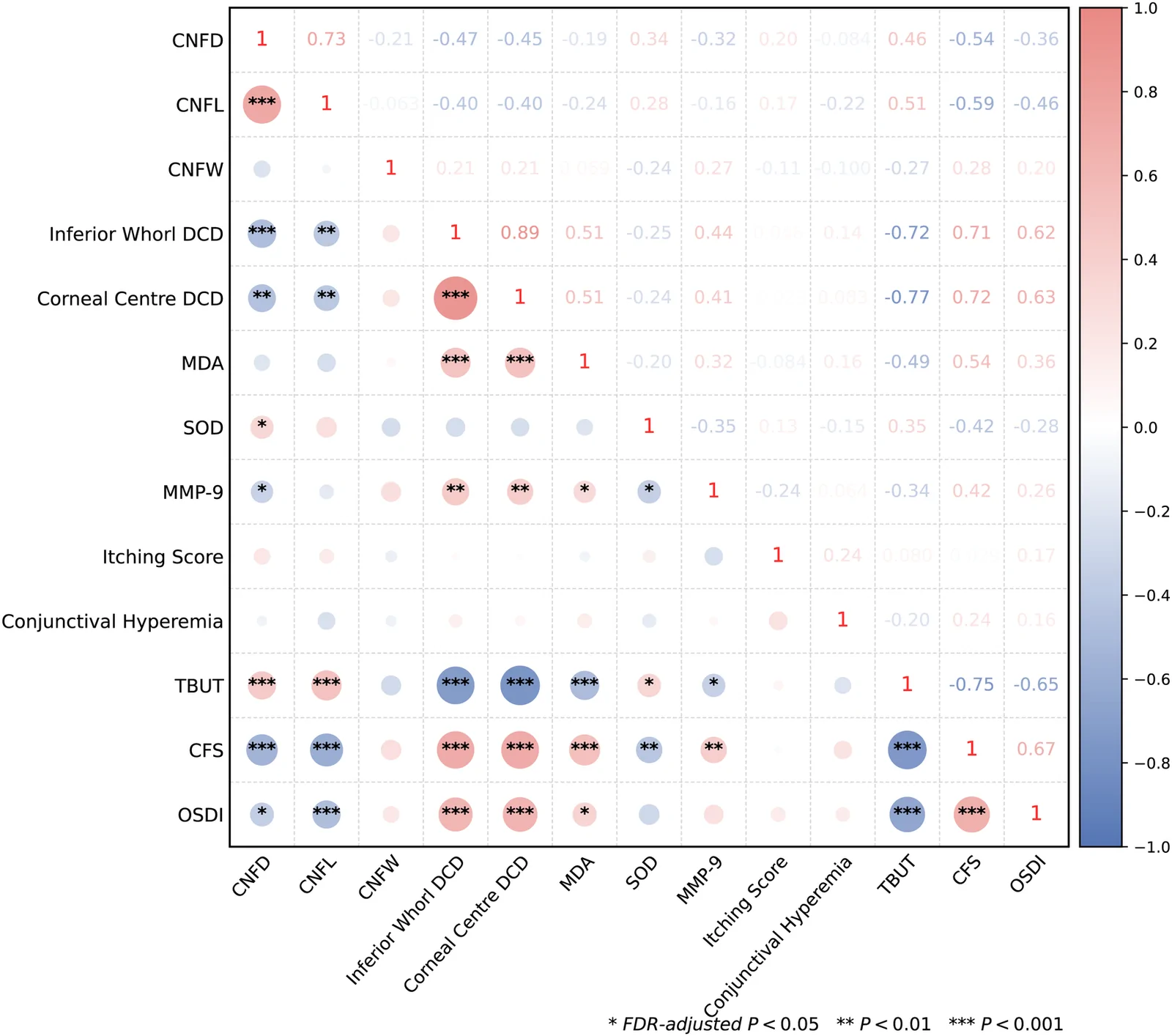
Exploratory correlation analysis of tear biomarkers, clinical ocular surface parameters, and IVCM findings. Spearman correlation heatmap illustrating the relationships among tear oxidative stress markers, MMP-9, clinical ocular surface parameters, and *in vivo* confocal microscopy (IVCM) findings in the pooled AC and AC + DE cohort (*n* = 60). Correlation coefficients are presented as Spearman's *ρ*. *P* values were adjusted for multiple comparisons using the Benjamini–Hochberg false discovery rate (FDR) procedure. Statistical significance is based on FDR-adjusted *P* values. AC, allergic conjunctivitis; AC + DE, allergic conjunctivitis-associated dry eye; MDA, malondialdehyde; SOD, superoxide dismutase; MMP-9, matrix metalloproteinase-9; OSDI, Ocular Surface Disease Index; TBUT, tear film break-up time; CFS, corneal fluorescein staining; CNFD, corneal nerve fiber density; CNFL, corneal nerve fiber length; DC, dendritic cell.*FDR-adjusted *P* < 0.05; **FDR-adjusted *P* < 0.01; ***FDR-adjusted *P* < 0.001.

Exploratory Spearman correlation analyses were performed in the pooled AC and AC + DE cohort (*n* = 60), and the results are shown in Figure 3. The correlation heatmap illustrates the overall relationships among tear oxidative stress markers, MMP-9, clinical ocular surface parameters, and IVCM findings. To account for multiple testing, *P* values were adjusted using the Benjamini–Hochberg false discovery rate (FDR) procedure.

Several associations were observed across the pooled cohort, including relationships between tear oxidative stress markers, MMP-9, tear film parameters, and selected IVCM measures. However, because the AC and AC + DE groups differed substantially in several of these variables, additional sensitivity analyses were performed. Spearman correlations were examined separately within the AC and AC + DE groups, and partial Spearman correlations were calculated after adjustment for group status. Most associations involving MDA, SOD, MMP-9, and ocular surface parameters were attenuated after adjustment for group status and were not consistently reproduced in the subgroup analyses (Supplementary Table S1). These findings suggest that some pooled correlations may partly reflect differences between the AC and AC + DE phenotypes and should therefore be interpreted cautiously.

### 3.6 Discriminative performance of tear biomarkers

**Figure 4:**
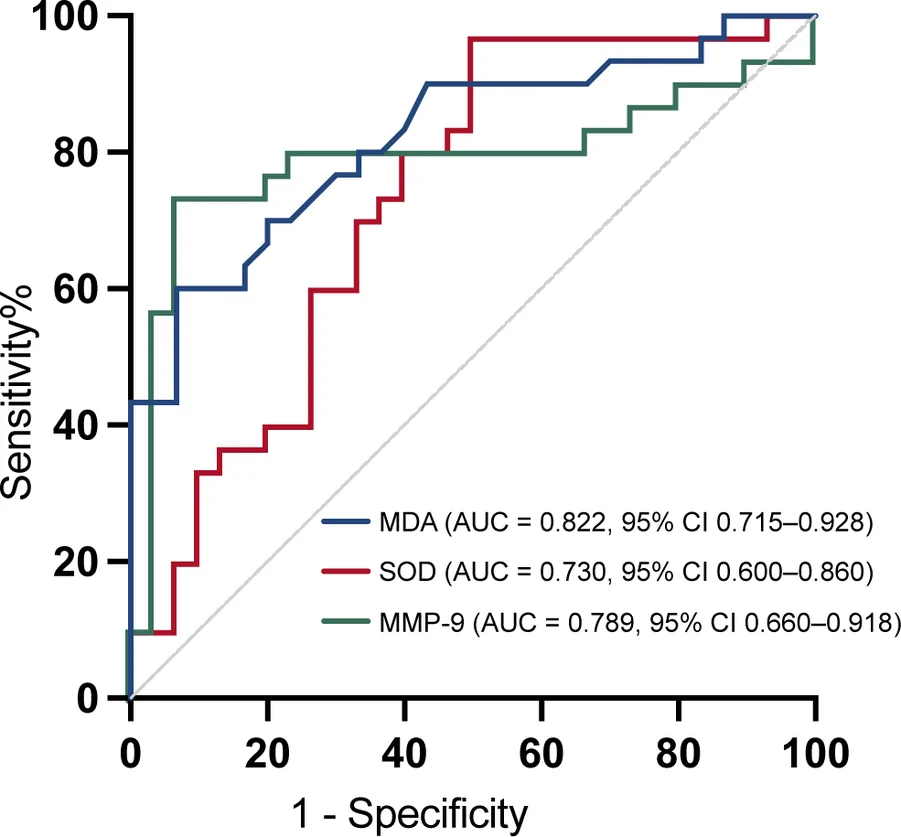
Receiver operating characteristic (ROC) curves of tear biomarkers for distinguishing allergic conjunctivitis-associated dry eye from allergic conjunctivitis. Receiver operating characteristic (ROC) curves of tear malondialdehyde (MDA) concentrations, tear superoxide dismutase (SOD) activity, and tear matrix metalloproteinase-9 (MMP-9) concentrations for distinguishing patients with allergic conjunctivitis-associated dry eye (AC + DE) from those with allergic conjunctivitis (AC). The area under the curve (AUC) was 0.822 (95% CI, 0.715–0.928) for MDA, 0.730 (95% CI, 0.600–0.860) for SOD, and 0.789 (95% CI, 0.660–0.918) for MMP-9. The diagonal dashed line represents the reference line (AUC = 0.5).

Receiver operating characteristic (ROC) curve analyses were performed to evaluate the ability of tear biomarkers to distinguish patients with AC + DE from those with AC alone (Figure 4). MDA showed the highest numerical AUC at 0.822 (95% CI, 0.715–0.928), followed by MMP-9 (AUC = 0.789; 95% CI, 0.660–0.918) and SOD (AUC = 0.730; 95% CI, 0.600–0.860). All three AUCs were statistically significant (all *P* < 0.01). Using the maximum Youden index, the optimal cutoff values were 2.42 nmol/mL for MDA, 407.2 U/mL for SOD, and 39.08 ng/mL for MMP-9, with sensitivities of 60.0%, 96.7%, and 73.3% and specificities of 93.3%, 50.0%, and 93.3%, respectively (Supplementary Table S2). Because no formal pairwise comparison of the ROC curves was performed, the numerical differences in AUC should not be interpreted as evidence that one biomarker was statistically superior to another.

## 4 Discussion

In this cross-sectional study, we compared clinical ocular surface findings, corneal neuroimmune alterations, and tear biomarkers related to oxidative stress and ocular surface barrier dysfunction among healthy controls, patients with allergic conjunctivitis (AC), and patients with allergic conjunctivitis-associated dry eye (AC + DE). Patients with AC + DE exhibited more severe ocular surface dysfunction, greater oxidative stress imbalance, higher tear MMP-9 concentrations, and more pronounced corneal neuroimmune alterations than patients with AC alone. Exploratory correlation analyses identified associations between oxidative stress markers, MMP-9, and selected tear film parameters, whereas receiver operating characteristic (ROC) analyses showed that MDA, SOD, and MMP-9 had discriminatory ability for AC + DE. Together, these observations suggest that oxidative stress, ocular surface barrier dysfunction, and corneal neuroimmune alterations are closely associated with the AC + DE phenotype and may help explain the marked variability in ocular surface involvement among patients with apparently similar allergic manifestations.

Although IgE-mediated allergic responses, mast cell activation, and T helper 2 (Th2)-dominant inflammation are well-established mechanisms underlying AC (20), these pathways primarily explain the initiation and persistence of allergic inflammation and do not fully account for the substantial interindividual differences in ocular surface damage. Increasing evidence has identified oxidative stress as an important link between chronic inflammation and tissue injury (7, 21–23). Persistent inflammatory stimulation promotes excessive production of ROS, leading to lipid peroxidation of cellular membranes, accumulation of end products such as MDA, impairment of antioxidant defense systems, and disruption of ocular surface redox homeostasis (7, 24, 25). In the present study, patients with AC + DE exhibited significantly higher tear MDA concentrations and lower SOD activity than patients with AC alone, indicating a greater degree of oxidative stress imbalance. This pattern is consistent with previous observations in dry eye disease and with the view that allergic inflammation and oxidative stress may coexist within a disturbed ocular surface microenvironment. These biochemical changes occurred alongside shorter TBUT, more severe corneal fluorescein staining, and higher OSDI scores, suggesting that oxidative stress imbalance may accompany greater ocular surface dysfunction and symptom burden in the AC + DE phenotype (23, 26).

Maintenance of the ocular surface barrier is essential for preserving tear film homeostasis and immune balance (13). MMP-9, a well-recognized biomarker associated with ocular surface barrier dysfunction, degrades epithelial tight junction proteins, including ZO-1, occludin, and claudins, thereby increasing epithelial permeability, destabilizing the tear film, and amplifying ocular surface inflammation (27–29). Previous studies have reported higher tear MMP-9 concentrations in patients with AC-associated dry eye than in those with dry eye alone or healthy individuals and have suggested that elevated MMP-9 may contribute to epithelial barrier disruption (30–33). However, factors associated with increased MMP-9 expression in AC remain incompletely understood. In our study, tear MMP-9 concentrations increased progressively from the HC group to the AC group and the AC + DE group. In the pooled cohort, MMP-9 was positively correlated with MDA and negatively correlated with SOD activity, suggesting a potential relationship between oxidative stress and MMP-9 elevation. Experimental studies have shown that oxidative stress can induce MMP-9 expression through activation of inflammatory signaling pathways, including NF-*κ*B, MAPK, and AP-1, thereby contributing to ocular surface barrier dysfunction (7, 34). MMP-9 was also negatively correlated with TBUT in the pooled analysis, consistent with previous reports linking elevated MMP-9 to reduced tear film stability (35–37). When considered together with the shorter TBUT and more severe corneal fluorescein staining observed in the AC + DE group, these findings are consistent with a relationship among MMP-9 elevation, tear film instability, and ocular surface epithelial damage. However, sensitivity analyses indicated that some of the pooled correlations were attenuated after accounting for AC versus AC + DE group status, suggesting that between-group differences may partly contribute to these associations.

IVCM revealed more pronounced reductions in corneal nerve parameters together with increased DC density in patients with AC + DE, indicating alterations in the corneal neuroimmune microenvironment. These changes were accompanied by shorter TBUT and more severe corneal fluorescein staining, suggesting that corneal nerve abnormalities may be associated with greater ocular surface dysfunction. Beyond their sensory function, corneal nerves play essential roles in tear secretion, blink reflexes, epithelial repair, and local neuroimmune regulation, all of which are critical for maintaining ocular surface homeostasis (10, 38). Damage to the corneal nerve plexus may therefore impair tear secretion, reduce tear film stability, delay epithelial healing, and contribute to persistent ocular surface dysfunction. Previous IVCM studies have likewise demonstrated reduced corneal nerve density together with increased DC density in dry eye disease, indicating that neural alterations and local immune activation may coexist during disease progression (39–44). Corneal nerve remodeling is likely to reflect the combined influence of chronic inflammation, oxidative stress, tear film instability, neurotrophic support, immune cell activation, and other changes in the local ocular surface microenvironment (38, 45), rather than being attributable to a single inflammatory pathway or biomarker. Given the cross-sectional nature of the present study, these observations should be interpreted as associations rather than evidence of temporal or causal relationships.

This study has several limitations. First, its single-center cross-sectional design and relatively small sample size limit causal inference and the generalizability of the findings. Second, some pooled correlations were attenuated after adjustment for AC versus AC + DE group status, suggesting a potential contribution of between-group differences. Third, only four participants were younger than 18 years, and the applicability of the OSDI to younger populations is less well established. In addition, healthy controls did not routinely undergo allergy testing; therefore, asymptomatic sensitization could not be completely excluded. Finally, only representative oxidative stress and barrier-related biomarkers were evaluated, without direct assessment of ROS generation, tight junction proteins, or related signaling pathways. Larger multicenter longitudinal studies and mechanistic investigations are needed to validate these findings and clarify the underlying mechanisms.

## 5 Conclusions

In conclusion, patients with AC + DE exhibited greater tear oxidative stress imbalance, elevated MMP-9 levels, impaired tear film function, and more pronounced corneal neuroimmune alterations than patients with AC alone. These findings suggest that oxidative stress, MMP-9 elevation, ocular surface dysfunction, and neuroimmune alterations may be interconnected within the AC + DE phenotype. This potential pathological framework may provide insight into the heterogeneous ocular surface manifestations of AC, although longitudinal and mechanistic studies are needed to clarify the temporal and causal relationships among these processes.

## Data Availability

The raw data supporting the conclusions of this article will be made available by the authors, without undue reservation.
