# Supplementary material for: Ocular surface oxidative stress, MMP-9, and corneal neuroimmune alterations in allergic conjunctivitis with dry eye: a cross-sectional study
Source: Front Med (Lausanne). 2026 Sep 8;13:1942693. doi: 10.3389/fmed.2026.1942693 (PMC13593414; doi:10.3389/fmed.2026.1942693)
Supplement: Supplementary file 1 [file Table1.docx]

**Supplementary Table S1. Sensitivity analyses of selected correlations in patients with allergic conjunctivitis**

| **Variables** | **Pooled ρ** | **FDR-adjusted P** | **AC ρ** | **FDR-adjusted P** | **AC+DE ρ** | **FDR-adjusted P** | **Group-adjusted partial ρ** | **FDR-adjusted P** |
| --- | --- | --- | --- | --- | --- | --- | --- | --- |
| MDA vs MMP-9 | 0.319 | 0.028 | 0.036 | 0.984 | 0.127 | 0.837 | 0.097 | 0.821 |
| SOD vs MMP-9 | −0.346 | 0.017 | −0.255 | 0.841 | −0.144 | 0.779 | −0.189 | 0.769 |
| MMP-9 vs TBUT | −0.341 | 0.018 | 0.373 | 0.505 | 0.036 | 0.937 | 0.207 | 0.672 |
| MMP-9 vs CNFL | −0.162 | 0.267 | 0.230 | 0.841 | 0.146 | 0.779 | 0.156 | 0.821 |
| MDA vs TBUT | −0.486 | <0.001 | −0.176 | 0.841 | 0.216 | 0.702 | 0.014 | 0.976 |
| MDA vs CFS | 0.537 | <0.001 | 0.379 | 0.505 | −0.051 | 0.937 | 0.203 | 0.672 |
| MDA vs OSDI | 0.362 | 0.012 | −0.032 | 0.984 | −0.193 | 0.716 | −0.117 | 0.821 |
| MMP-9 vs CNFD | −0.321 | 0.028 | −0.186 | 0.841 | 0.097 | 0.882 | −0.026 | 0.976 |
| MMP-9 vs CFS | 0.416 | 0.003 | 0.026 | 0.984 | −0.035 | 0.937 | 0.020 | 0.976 |

Note. Spearman's rank correlation analyses were performed in the pooled AC and AC+DE cohort (n = 60) and separately within the AC (n = 30) and AC+DE (n = 30) groups. Partial Spearman correlation analyses were additionally performed in the pooled cohort after adjustment for group status (AC vs AC+DE). Values are presented as Spearman's ρ or partial Spearman's ρ. P values were adjusted for multiple comparisons using the Benjamini–Hochberg false discovery rate (FDR) procedure across all 78 pairwise comparisons within each analysis. AC, allergic conjunctivitis; AC+DE, allergic conjunctivitis-associated dry eye; MDA, malondialdehyde; SOD, superoxide dismutase; MMP-9, matrix metalloproteinase-9; TBUT, tear film break-up time; CFS, corneal fluorescein staining; OSDI, Ocular Surface Disease Index; CNFD, corneal nerve fiber density; CNFL, corneal nerve fiber length.
