# Supplementary material for: Ocular surface oxidative stress, MMP-9, and corneal neuroimmune alterations in allergic conjunctivitis with dry eye: a cross-sectional study
Source: Front Med (Lausanne). 2026 Sep 8;13:1942693. doi: 10.3389/fmed.2026.1942693 (PMC13593414; doi:10.3389/fmed.2026.1942693)
Supplement: Supplementary file 2 [file Table2.docx]

**Supplementary Table 2. ROC characteristics of tear biomarkers for distinguishing AC+DE from AC alone**

| **Biomarker** | **AUC** | **95% CI** | **Optimal cutoff** | **Sensitivity (%)** | **Specificity (%)** | **Youden index** |
| --- | --- | --- | --- | --- | --- | --- |
| MDA | 0.822 | 0.715–0.928 | ≥2.42 | 60.0 | 93.3 | 0.533 |
| SOD | 0.730 | 0.600–0.860 | ≤407.2 | 96.7 | 50.0 | 0.467 |
| MMP-9 | 0.789 | 0.660–0.918 | ≥39.08 | 73.3 | 93.3 | 0.667 |

Note. Optimal cutoff values were determined by maximizing the Youden index (sensitivity + specificity − 1). For MDA and MMP-9, values at or above the cutoff were classified as AC+DE; for SOD, values at or below the cutoff were classified as AC+DE. AUC, area under the receiver operating characteristic curve; CI, confidence interval; AC, allergic conjunctivitis; AC+DE, allergic conjunctivitis-associated dry eye; MDA, malondialdehyde; SOD, superoxide dismutase; MMP-9, matrix metalloproteinase-9.
